# Visualization of microRNA-21 Dynamics in Neuroblastoma Using Magnetic Resonance Imaging Based on a microRNA-21-Responsive Reporter Gene

**DOI:** 10.3389/fonc.2021.747305

**Published:** 2021-11-05

**Authors:** Guangcheng Bao, Jun Sun, Helin Zheng, Jingxin Hou, Jie Huang, Jie Wei, Yuanqiao Fu, Jiawen Qiu, Xuefeng Zou, Bin Xiang, Jinhua Cai

**Affiliations:** ^1^ Department of Radiology, Children’s Hospital of Chongqing Medical University, Chongqing, China; ^2^ Ministry of Education Key Laboratory of Child Development and Disorders, Chongqing, China; ^3^ Key Laboratory of Pediatrics in Chongqing, Chongqing, China; ^4^ Chongqing International Science and Technology Cooperation Center for Child Development and Disorders, Chongqing, China; ^5^ Department of Radiology, Chongqing University Central Hospital, Chongqing, China; ^6^ Department of Radiology, Second Affiliated Hospital of Chongqing Medical University, Chongqing, China; ^7^ Guizhou Provincial Key Laboratory of Computational Nano-Material Science, Guizhou Education University, Guiyang, China; ^8^ Chemistry and Chemical Engineering, Chongqing University, Chongqing, China

**Keywords:** microRNA-21, reporter gene, ferritin heavy chain, magnetic resonance imaging, neuroblastoma

## Abstract

**Background:**

MicroRNAs (miRs) have been shown to be closely associated with the occurrence and development of tumors and to have potential as diagnostic and therapeutic targets. The detection of miRs by noninvasive imaging technology is crucial for deeply understanding their biological functions. Our aim was to develop a novel miR-21-responsive gene reporter system for magnetic resonance imaging (MRI) visualization of the miR-21 dynamics in neuroblastoma.

**Methods:**

The reporter gene ferritin heavy chain (FTH1) was modified by the addition of 3 copies of the sequence completely complementary to miR-21 (3xC_miR-21) to its 3’-untranslated region (3’ UTR) and transduced into SK-N-SH cells to obtain SK-N-SH/FTH1-3xC_miR-21 cells. Then, the antagomiR-21 was delivered into cells by graphene oxide functionalized with polyethylene glycol and dendrimer. Before and after antagomiR-21 delivery, FTH1 expression, MRI contrast and intracellular iron uptake were assayed *in vitro* and *in vivo*.

**Results:**

In the SK-N-SH/FTH1-3xC_miR-21 cells, FTH1 expression was in an “off” state due to the combination of intratumoral miR-21 with the 3’ UTR of the reporter gene. AntagomiR-21 delivered into the cells bound to miR-21 and thereby released it from the 3’ UTR of the reporter gene, thus “switching on” FTH1 expression in a dose-dependent manner. This phenomenon resulted in intracellular iron accumulation and allowed MRI detection *in vitro* and *in vivo*.

**Conclusion:**

MRI based on the miR-21-responsive gene reporter may be a potential method for visualization of the endogenous miR-21 activity in neuroblastoma and its response to gene therapy.

## Introduction

MicroRNAs (miRs) are endogenous single-stranded small-molecule RNAs that are approximately 20-25 nucleotides long, do not encode proteins, and have a variety of important regulatory functions in cells (1). miRs can target and bind the 3’-untranslated regions (3’ UTRs) of specific messenger RNAs (mRNAs) and induce their translational repression or degradation, thereby decreasing the expression of proteins encoded by the targeted mRNAs. This novel mode of post-transcriptional gene regulation plays an important role in extensive biological processes, such as cell growth, differentiation, apoptosis, tumorigenicity and chemoresistance (2, 3).

Since the first report of the role of miRs in oncogenesis (4), many miRs involved in cancer development have been identified. Among these, some function as onco-miRs by promoting tumor proliferation and angiogenesis, while some are associated with tumor inhibition (5–7). The different functions of these miRs may be related to the type and stage of cancer development. Currently, the expression profiles of miRs and their potential targets have been widely studied in breast (8), lung (9), prostate (10), colon (11), gastric (12) and ovarian (13) cancer. Among various cancer-associated miRs, miR-21 has attracted substantial attention as a potential diagnostic and therapeutic cancer target since it has been reported to be the only carcinogenic miR that is overexpressed in several types of solid tumors, including neuroblastoma, glioblastoma, breast cancer, colorectal cancer and prostate cancer (4, 8, 10, 11, 14). On the other hand, a chemically modified antisense RNA oligonucleotide has been used to target and repress the activity of miR-21, thereby inhibiting tumor development (15, 16).

Considering the potential of miR to serve as a diagnostic and therapeutic biomarker in cancer, investigations of the biological properties and expression patterns of miRs are crucial. The conventional methods for detecting endogenous miRs include northern blotting (17), quantitative reverse-transcriptase polymerase chain reaction (qRT-PCR) (9), small RNA sequencing (18) and microarray (19). However, these methods require cell lysis and are not suitable for detecting miR expression in living cells. Therefore, the development of noninvasive methods is imperative for elucidating the expression and regulation of miRs *in vivo*.

At present, several noninvasive imaging techniques, such as optical imaging, radionuclide imaging and magnetic resonance imaging (MRI), have been applied to detect biological processes *in vivo* at the cellular or molecular level (20, 21). Of these imaging methods, MRI is thought to be ideal due to its advantages, including deep tissue penetration, high tissue resolution, repeatable examination and nonrequirement for radiation. Molecular MRI usually relies on a reporter gene, which can be integrated into the cellular genome and allows for the long-term monitoring of biological events. Among various MRI reporter genes, ferritin heavy chain 1 (FTH1) has attracted substantial attention because its expression can induce the effective cellular uptake of endogenous iron, which leads to sensitive MRI signal contrast (22–28). In our previous studies, we successfully monitored mesenchymal stem cells with FTH1-based MRI *in vitro* and *in vivo* (24, 29). Considering the advantages of FTH1 as an endogenous reporter gene, it is worth investigating the feasibility of FTH1-based MRI to visualize miR activities, and this method could serve as a noninvasive imaging modality for miR detection.

**Figure 1:**
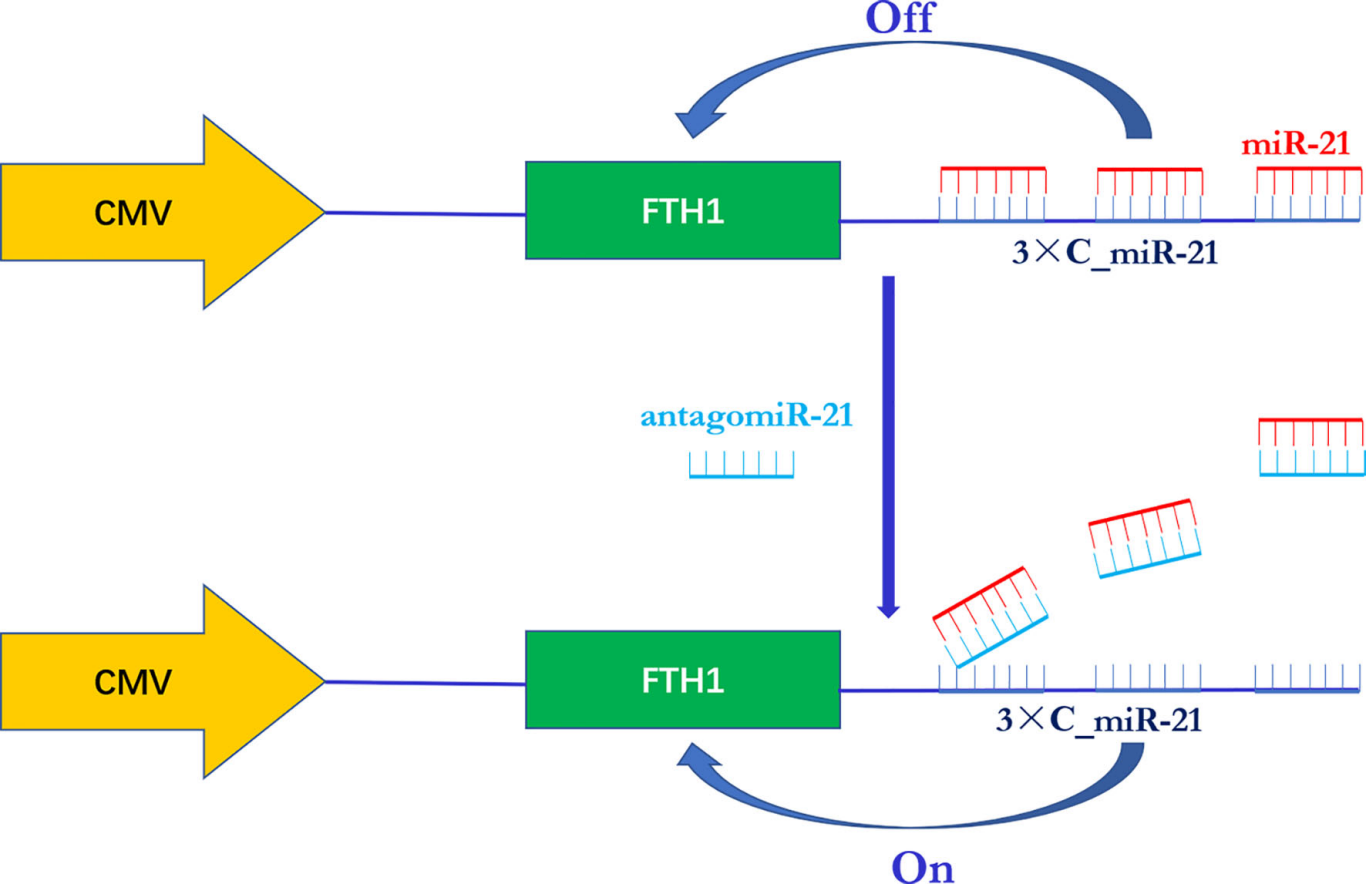
Schematic illustration of the miR-21-responsive FTH1 gene reporter system. The reporter gene FTH1 is modified by the addition of 3 copies of the sequence completely complementary to miR-21 (3xC_miR-21) to its 3’-untranslated region (3’ UTR). In the presence of miR-21, FTH1 expression is switched “off” due to the combination of miR-21 with the 3’ UTR of the reporter gene. Once an exogenous antisense oligonucleotide (antagomiR-21) is administered, it binds to miR-21 in a competitive manner and releases miR-21 from the 3’ UTR of FTH1, thereby switching “on” reporter gene expression.

In this study, we designed a novel miR-21-responsive FTH1 expression system in which the reporter gene FTH1 was modified by the addition of 3 copies of the sequence completely complementary to miR-21 (3xC_miR-21) to its 3’ UTR. When the reporter gene system was transduced into neuroblastoma cells, in which miR-21 functions as an oncogenic biomarker, its expression was expected to be switched “off” due to the combination of intratumoral miR-21 with the 3’ UTR of the reporter gene. An exogenous antisense oligonucleotide (antagomiR-21) was then delivered into the cells and expected to bind with miR-21 in a competing manner, thereby releasing miR-21 from the FTH1 3’ UTR and switching “on” the reporter gene expression (**Figure 1**). With the reporter gene system, we aimed to provide a noninvasive and efficient imaging modality for visualizing the activity of miR-21 and its response to gene therapy.

## Materials and Methods

### Construction of the miR-21-Responsive FTH1 Expression Vector

The cDNA of human FTH1 (Gene ID: 2495) was obtained *via* polymerase chain reaction (PCR) amplification using the following primers: forward, AACCGTCAGATCGCACCGGTGCCACCATGACGACCGCGTCCACCTC and reverse, TCCTTGTAGTCCATGAATTCGCTTTCATTATCACTGTCTC. The synthesized FTH1 cDNA was subcloned into the EcoR I and Sma I restriction endonuclease sites of the plasmid pLVX-mCMV-ZsGreen1-Puro to obtain the recombinant plasmid vector pLVX-mCMV-ZsGreen1-Puro-FTH1 (pLV-FTH1). Then, the DNA oligonucleotides containing 3xC_miR-21 (sense 5’ GTAGCTTATCAGACTGATGTTGATAGTAGTAGCTTATCAGACTGATGTTGATAGTAGTAGCTTATCAGACTGATGTTGA 3’; antisense 5’ TCAACATCAGTCTGATAAGCTACTACTATCAACATCAGTCTGATAAGCTACTACTATCAACATCAGTCTGATAAGCTAC 3’) were synthesized and ligated into pLV-FTH1 to yield the final lentiviral vector plasmid containing 3xC_miR-21 (pLV-FTH1-3xC_miR-21). After verification by PCR analysis and DNA sequencing, the plasmid pLV-FTH1-3xC_miR-21 was cotransfected together with psPAX2 and pMD2.G into 293 T packaging cells (Invitrogen, Carlsbad, CA, USA) to produce the lentivirus containing FTH1-3xC_miR-21.

### Cell Culture and Establishment of Stable Transgenic Cells

The wild-type human neuroblastoma cell line (SK-N-SH/WT) was provided by the Chongqing Institute of Pediatric Medicine Research and cultured in Dulbecco’s modified Eagle’s medium (DMEM; Gibco, Grand Island, NY, USA) containing 10% fetal bovine serum (FBS; Gibco, Grand Island, NY, USA) and 0.5% streptomycin at 37°C in a moist environment containing 5% CO2. According to the lentivirus transfection protocol, cells at 30-40% confluence were transfected with the FTH1-3xC_miR-21 lentivirus and screened with 10 μg/ml puromycin. Finally, the surviving clones were isolated and named SK-N-SH/FTH1-3xC_miR-21 cells, with SK-N-SH/WT cells and SK-N-SH/FTH1 (without 3xC_miR-21) cells serving as controls.

### Assessment of Cell Growth

To investigate the effect of gene transduction on cell viability, cell growth was assayed by Cell Counting Kit-8 (CCK-8) colorimetry (Beyotime, Nanjing, Jiangsu, China). SK-N-SH/WT, SK-N-SH/FTH1 and SK-N-SH/FTH1-3×C_miR-21 cells were cultured in 96-well plates for 72 h, and the relative changes in the three cell lines were determined.

### AntagomiR-21 Transfection Into Cells

The antisense RNA oligonucleotide, antagomiR-21, was provided by GenePharma, Shanghai, China. The sequence of antagomiR-21 was as follows: 5′-GUCAACAUCAGUCUG AUA AGCUA-3.

For improvement of the transfection efficiency of antagomiR-21, graphene oxide (GO) modified with polyethylene glycol (PEG) and polyamidoamine dendrimer (GO-PEG-dendrimer) was used as gene delivery carrier. GO was synthesized by previously reported methods (30–35), and the detailed protocol for synthesizing GO-PEG-dendrimer and its characterization are shown in the **Supplementary Material**.

The efficiency of the GO-PEG-dendrimer to deliver antagomiR-21 could be affected by the GO/PEG ratio. Too little GO could reduce its capacity of combining with antagomir-21 and lead to a low transducing efficiency, while too little PEG could decrease the biocompatibility which also result a low transducing efficiency. To obtain the optimal structure of GO-PEG-dendrimer for efficiently delivering anagomiR-21 into SK-N-SH cells, we compared the transducing efficiency of the GO-PEG-dendrimer complex at various GO/PEG ratios. The GO-PEG-dendrimer at GO/PEG molar ratios of 5:1, 1:5 and 1:1 was mixed with 30 pmol of anagomiR-21 modified by the fluorescence dye Cy3 (anagomiR-21-Cy3) to prepare GO-PEG-dendrimer/anagomiR-21-Cy3 complexes. Then, 2 μL of siRNA-Mate (GenePharma, Shanghai, China), a commonly used small-molecule RNA transfection agent, was mixed with 30 pmol of antagomiR-21-Cy3 to obtain the siRNA-Mate/antagomiR-21-Cy3 complex as a control. The obtained complexes were incubated with SK-N-SH cells for 6 h, washed 3 times, fixed with 4% formaldehyde and stained with 4’,6-diamidino-2-phenylindole (DAPI). Fluorescence images were obtained by a fluorescence microscope (Nikon A1R, Tokyo, Japan).

A CCK-8 assay was used to compare the cytotoxicity of the GO-PEG-dendrimer complexes and siRNA-Mate. SK-N-SH cells were divided into two groups, cultured in 96-well plates at a density of 6 × 10^3^ cells per well, and cocultured with the GO-PEG-dendrimer (GO/PEG ratio 1:1) or siRNA-Mate at equal concentrations for 72 h. Then, the absorbance was measured every 12 h after the addition of CCK-8 reagent.

### Western Blot and qRT-PCR Assays

FTH1 expression in SK-N-SH/WT, SK-N-SH/FTH1 and SK-N-SH/FTH1-3×C_miR-21 cells was detected by qRT-PCR. Briefly, total RNA was extracted using a TaKaRa MiniBEST Universal RNA Extraction Kit (TaKaRa^®^, Kyoto, Japan) and then reverse transcribed into cDNA using HiScript^®^II Q RT SuperMix (Vazyme, Nanjing, China) for qPCR, which was carried out using AceQ^®^ qPCR SYBR Green Master Mix (Vazyme) on an AB Step One plus Real Time PCR System (Applied Biosystems AB, Waltham Mass, USA). GAPDH expression served as the quantitative internal control for FTH1, and each experiment was performed in triplicate. The primers for GAPDH and FTH1 detection were as follows: GAPDH-F AGAAGGCTGGGGCTCATTTG and GAPDH-R AGGGGCCATCCACAGTCTTC; FTH1-F GGAATTCATGACGACCGCGTCCAC and FTH1-R CCCCGGGAGCTTTCATTATCACTGTCTCCC.

Simultaneously, proteins were extracted from the three cell lines and prepared for Western blot (WB) analysis. Briefly, the cells were cleaved in cleavage buffer (Sigma, St. Louis, Missouri, USA) containing protease inhibitors, phosphatase inhibitors and 100 mM benzene sulfonyl fluoride, and the total protein concentration in the sample was determined by the BCA method. Thirty micrograms of protein from each sample was then added to a 12% sodium dodecyl sulfate-polyacrylamide gel and transferred onto a polyvinylidene fluoride (PVDF) membrane (Bio-Rad, California, USA). For detection of FTH1, the membrane was blocked with 5% bovine serum albumin (BSA; Beyotime) for 1 h and then incubated with a primary antibody that specifically recognized FTH1 (rabbit anti-FTH1, 1:1000; Abcam, Cambridge, England) or GAPDH (rabbit anti-FTH1, Abcam) overnight at 4°C. The membrane was then washed with Tris-buffered saline containing Tween-20 and incubated with a secondary antibody (goat anti-rabbit IgG, 1:5000; Sigma) for 2 h. The protein bands were observed using an enhanced chemiluminescence kit (Sigma). The relative expression of FTH1 in SK-N-SH/FTH1-3C×_miR-21 cells was normalized to that of GAPDH, and semiquantitative analysis was carried out based on the band strength.

For determination of the correlation between FTH1 expression and the amount of antagomiR-21, SK-N-SH/FTH1-3×C_miR-21 cells transfected with antagomiR-21 at different concentrations (0, 10, 10, 20, 30, 40, 40, 50, or 60 nmol/L) were cultured for 24 h, and cellular proteins were collected and prepared for WB analysis.

### MRI of Cell Pellets

To assess the effect of FTH1 expression on cellular iron transfer, we cultured SK-N-SH/FTH1-3C×_miR-21 cells in medium containing 500 μmol/L ferric ammonium citrate (FAC; Sigma) and then transfected them with antagomiR-21 at the same concentration gradient as that used for the WB experiment. After 24 h, all the treated cells were washed thoroughly with phosphate-buffered saline (PBS) to remove the free FAC, digested with ethylenediaminetetraacetic acid (EDTA), suspended in PBS, and then transferred into a 0.6-mL Eppendorf tube to prepare the cell phantom for MRI *in vitro*. The cellular phantom was imaged using a 7.0 T MR scanner (Bruker, Karlsruhe, Germany). The spin echo (SE) T2 weighted imaging (T2WI) parameters were as follows: time of repetition (TR), 2,500 ms; time of echo (TE), 35 ms; field of view (FOV), 120 mm×120 mm; slice thickness, 1 mm; slice interval, 0.1 mm. The parameters for the multiecho sequence were as follows: TR, 2,000 ms; TE, 8~200 ms with a step size of 8 ms (25-point T2 mapping); other parameters, including the FOV, matrix and slice thickness, matched those used for T2WI imaging. T2 maps were obtained by image postprocessing, and the R2 value was measured.

To evaluate FTH1 expression and its effect on iron transfer in groups of cells treated with or without antagomir-21, SK-N-SH/WT, SK-N-SH/FTH1 and SK-N-SH/FTH1-3×C_miR-21 cells were cultured with antagomir-21 at the optimal concentration and 500 μmol/L FAC for 24 h. Before and after antagomiR-21 treatment, the three cell lines were subjected to MRI examination.

### Intracellular Iron Detection and Quantification

Prussian blue staining and transmission electron microscopy (TEM) were performed to detect intracellular iron accumulation. SK-N-SH/WT, SK-N-SH/FTH1 and SK-N-SH/FTH1-3×C_miR-21 cells transfected with or without antagomir-21 were cultured together with 500 μmol/L FAC for 24 h. Then, the cells were harvested and subjected to Prussian blue staining and TEM examination according to our previous protocols (24). The intracellular accumulation of iron was observed under a light microscope (Nikon, Tokyo, Japan) or an H-7500 transmission electron microscope (Hitachi, Tokyo, Japan). For quantification of the intracellular iron content, 1×10^6^ cells per group were prepared according to the previous protocols (36). The iron concentration was measured using an atomic absorption spectrophotometer (Huaguang HG-960 2A, Shenyang, China). Each sample was measured 3 times. The concentration values are presented in units of pg/cell.

### *In Vivo* Experiments

The experimental animals were purchased from the Department of Medical Experimental Animals of Chongqing Medical University and raised at the Experimental Animal Center of our hospital. All animal protocols used in this study were reviewed and approved by the Animal Care and Use Committee of Chongqing Medical University, and the experimental procedures were performed in accordance with the National Institutes of Health guidelines. All efforts were made to minimize animal suffering.

The nude mice were divided into the following 3 groups: SK-N-SH/WT, SK-N-SH/FTH1 and SK-N-SH/FTH1-3C×_miR-21. A total of 2 ×10^6^ cells were inoculated subcutaneously into the right flanks of nude mice in each of the three groups to establish the transplanted tumor model of neuroblastoma. To enhance iron recruitment into cell xenografts, the animals were treated with FAC at a dose of 5 mg/L *via* their drinking water. When the tumors reached approximately 300 mm^3^ in size, the GO-PEG dendrimer/antagomiR-21 complex was administered *via* caudal vein injection at an antagomiR-21 dose of 30 nmol/g according to the manufacturer’s instructions. Before and 24 h after antagomiR-21 treatment, the animals were anesthetized and subjected to MRI examination. A 7.0 T MRI scanner was applied, and MR images were obtained by using the T2WI and multiecho sequences. The T2WI scanning parameters were as follows: TR, 2,500 ms; TE, 35 ms. The multiecho parameters were as follows: TR, 2,000 ms; TE, 8~200 ms with a step size of 8 ms (25-point T2 mapping); matrix, 380×300; FOV, 160 mm; and slice thickness, 1.2 mm. The R2 value was measured on T2 maps obtained by image postprocessing.

Immediately after MRI, the animals were sacrificed, and the masses were removed for histological examination. Prussian blue staining was also performed to detect iron accumulation in the tumors. In addition, the intratumoral iron content was quantified according to the protocols used for intracellular iron quantification and are presented in units of mg/g.

### Statistical Analysis

All data are expressed as the mean ± standard deviation. Statistical Package for the Social Sciences version 13.0 (SPSS Inc., Chicago, IL, USA) was used for the statistical analyses. One-way analysis of variance and the least significant difference method were used to compare differences among the groups. *P* values less than 0.05 were considered statistically significant.

## Results

### The miR-21-Responsive FTH1 Reporter Gene System and Its Effect on Cell Viability

The miR-21-responsive FTH1 gene reporter system, namely, FTH1-3×C_miR-21, which contained 3 copies of a sequence completely complementary to miR-21 at the 3’ UTR of the FTH1 gene, was successfully constructed. This reporter gene system was transduced into SK-N-SH cells, and SK-N-SH/FTH1-3×C_miR-21 cells were established.

**Figure 2:**
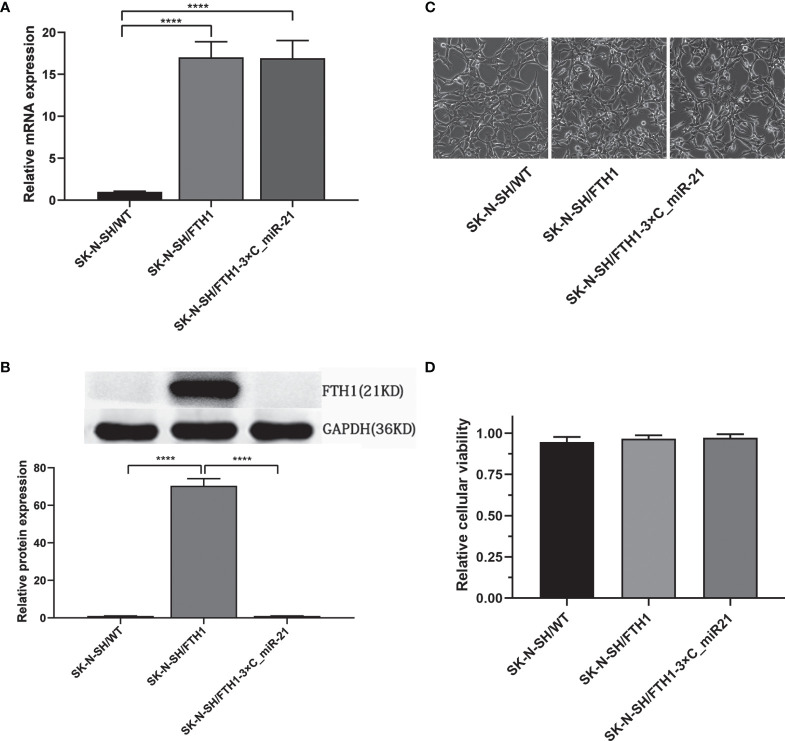
FTH1 expression and its effect on viability in the three groups of cells. The qRT-PCR results **(A)** showed that the mRNA expression levels of the FTH1 gene in the SK-N-SH/FTH1 and SK-N-SH/FTH1-3 × C_miR-21 groups was similar, and both groups had significantly higher levels than the SK-N-SH/WT group. The WB results **(B)** showed that compared with the SK-N-SH/WT group, the SK-N-SH/FTH1 group showed significantly increased FTH1 expression at the protein level, while the SK-N-SH/FTH1-3×C_miR-21 group did not show alterations in FTH1 expression. No morphological changes were observed among the SK-N-SH/WT, SK-N-SH/FTH1 and SK-N-SH/FTH1-3×C_miR-21 cells **(C)**. The CCK-8 results revealed no difference in cell viability among the three groups **(D)**. Three independent experiments were performed. **** indicates *P* < 0.0001.

To verify the activity of the reporter gene system, we evaluated FTH1 expression in the SK-N-SH/WT, SK-N-SH/FTH1 and SK-N-SH/FTH1-3×C_miR-21 cells by qRT-PCR and WB. The qRT-PCR results showed that the mRNA expression levels of the FTH1 gene in the SK-N-SH/FTH1 and SK-N-SH/FTH1-3×C_miR-21 groups were similar and both were significantly higher than that in SK-N-SH/WT group (*P* < 0.0001). The WB results showed that the protein level of FTH1 was increased significantly (*P <*0.0001) in the SK-N-SH/FTH1 group but not altered significantly in the SK-N-SH/FTH1-3×C_miR-21 group compared with the SK-N-SH/WT group (**Figures 2A, B**). These results indicated that miR-21 inhibited FTH1 expression at the post-transcriptional level.

To assess the safety of the reporter gene system, cell viability was evaluated. Morphologically, no changes were observed among the SK-N-SH/WT, SK-N-SH/FTH1 and SK-N-SH/FTH1-3×C_miR-21 cells (**Figure 2C**). The CCK-8 assay results also showed no significant differences in cell proliferation among the three groups (**Figure 2D**).

### Gene Transfection Efficiency and Cytotoxicity of the GO-PEG Dendrimer

**Figure 3:**
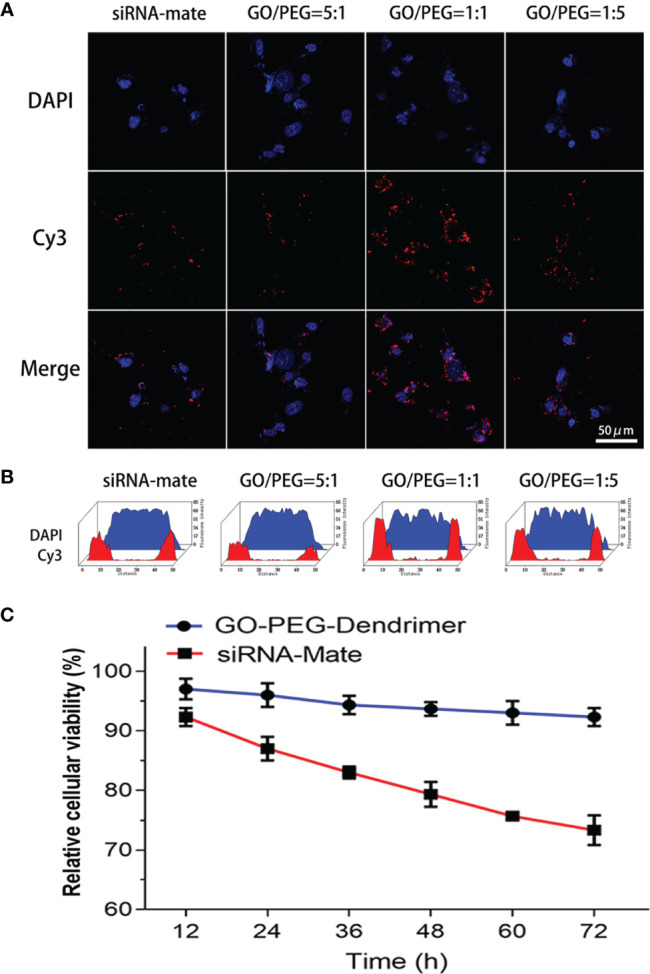
Transfection efficiency and cytotoxicity of the GO-PEG-dendrimer. Both the immunofluorescence staining **(A)** and fluorescence intensity quantification **(B)** results show that among the three GO-PEG-dendrimer structures with different GO/PEG ratios, the structure with a GO/PEG ratio of 1:1 had the highest transfection efficiency and was more efficient than the commonly used transfection reagent siRNA-Mate. The other two structures with GO/PEG ratios of 5:1 and 1:5 had transfection efficiencies that were similar to that of the siRNA-Mate. The CCK-8 assay **(C)** showed that the GO-PEG-dendrimer was much less cytotoxic than siRNA-Mate during the 72 h of coculture with SK-N-SH cells.

Immunofluorescence staining and fluorescence intensity quantification showed that the GO-PEG dendrimer with various GO/PEG ratios differentially introduced antagomir-21 into cells. Among the three structures with GO/PEG ratios of 1:5, 1:1 and 5:1, the GO-PEG dendrimer with a GO/PEG ratio of 1:1 was the most efficient and was more efficient than the commonly used transfection reagent siRNA-Mate (**Figures 3A, B**).

To study the cytotoxicity of the GO-PEG-dendrimer, we evaluated the survival rate of SK-N-SH cells. As shown in **Figure 3C**, the cytotoxicity of the GO-PEG-dendrimer was much lower than that of siRNA-Mate, indicating that GO-PEG-dendrimer is safe as a gene delivery vector.

### Dose-Dependent FTH1 Expression Regulated by AntagomiR-21

**Figure 4:**
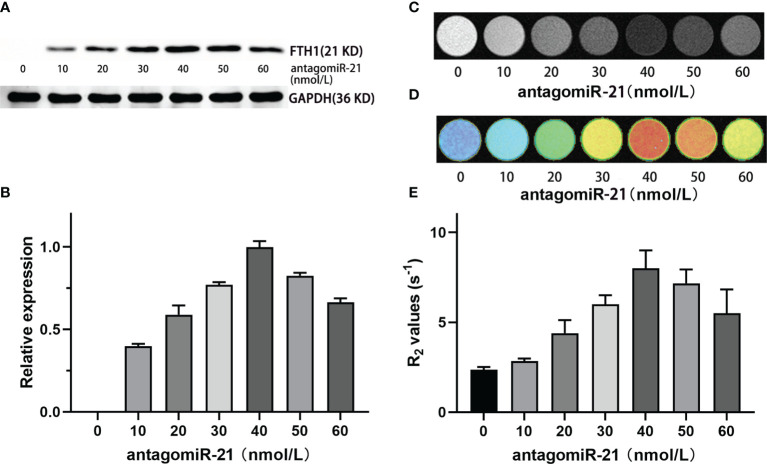
Regulation of FTH1 expression by antagomiR-21. WB **(A)** and gray-scale **(B)** results show that the delivery of antagomiR-21 into cells reversed FTH1 expression in a dose-dependent manner. In the absence of antagomiR-21, no obvious FTH1 expression was detected in SK-N-SH/FTH1-3 × PT cells in which miR-21 suppressed the reporter gene by binding with the completely complimentary sequence of 3×C_miR-21. After transfection with antagomir-21, the expression of FTH1 increased gradually as the antagomir-21 concentration increased, peaking at 40 nmol/L, and then slightly decreased as the antagomiR-21 concentration continuously increased. The cellular MRI T2WI **(C)**, T2 map **(D)** and R2 measurements **(E)** in cells treated with different concentrations of antagomiR-21 were consistent with the WB results.

To investigate the ability of antagomiR-21 to knock down miR-21 and reverse FTH1 expression in SK-N-SH/FTH1-3×C_miR-21 cells, we carried out WB to evaluate FTH1 expression in the cells treated with different concentrations of antagomir-21. AntagomiR-21 delivery into cells reversed FTH1 expression in a dose-dependent manner. In the absence of antagomiR-21, no obvious FTH1 expression was detected in the SK-N-SH/FTH1-3×C_miR-21 cells in which miR-21 suppressed the reporter gene by binding with the completely complimentary sequence of 3×C_miR-21. After transfection with antagomir-21, FTH1 expression increased gradually as the antagomir-21 concentration increased, peaking at 40 nmol/L antagomir-21, and then slightly decreased as the antagomiR-21 continuously increased (**Figures 4A, B**). The cellular MRI T2WI and R2 measurements of cells treated with different concentrations of antagomiR-21 were consistent with the WB results (**Figures 4C–E**).

### Cellular MRI Contrast Produced by the Regulatory Expression of FTH1

**Figure 5:**
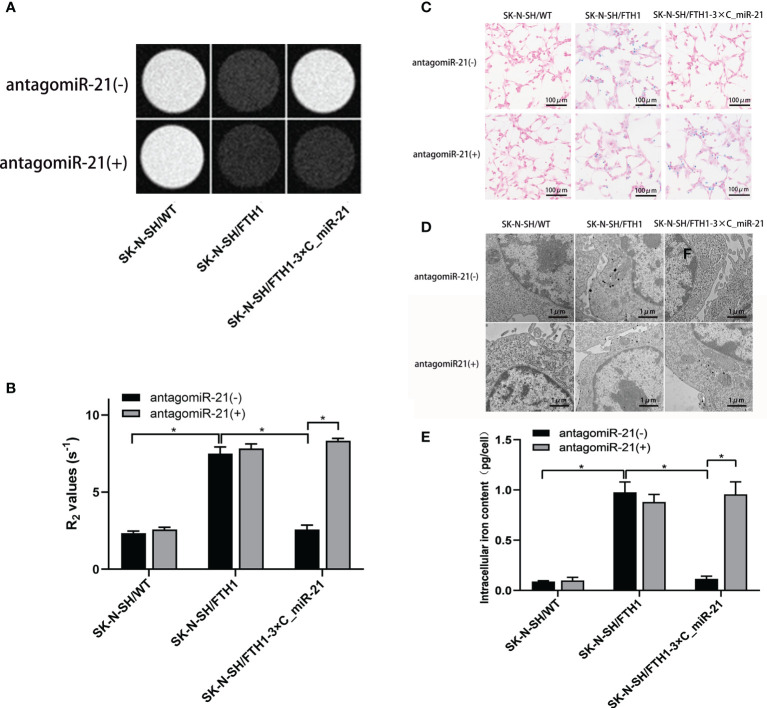
Cellular MRI and intracellular iron detection. MRI imaging shows a significant T2WI signal reduction **(A)** and R2 value increase **(B)** in the SK-N-SH/FTH1-3×C_miR-21 cells after antagomiR-21 transfection compared with that prior to antagomiR-21 treatment. In the SK-N-SH/WT and SK-N-SH/FTH1 cells, however, there were no differences in the MRI signals or R2 values before and after antagomiR-21 treatment. The Prussian blue staining **(C)** and TEM **(D)** results revealed that before antagomiR-21 transfection, large amounts of iron particles were detected in the cytoplasm of only SK-N-SH/FTH1 cells, while iron accumulation was not observed in SK-N-SH/WT or SK-N-SH/FTH1-3×C_miR-21 cells. After antagomiR-21 treatment, SK-N-SH/FTH1-3×C_miR-21 cells exhibited obvious iron accumulation compared with that prior to antagomiR-21 treatment. In the other two groups of cells, however, there was almost no change in the iron accumulation before and after antagomiR-21 treatment. Further iron content quantification **(E)** shows the same results as observed in Prussian blue staining and TEM. Three independent experiments were performed. * indicates *P* < 0.05.

The expression of FTH1 may increase the ability of cells to internalize and store iron ions, thereby decreasing the MRI signal. To assess the feasibility of using MRI to detect miR-21 activity regulated by antagomiR-21, we first determined the optimal concentration (40 nmol/L) at which antagomiR-21 induced the most obvious MRI signal change. Then, MRI was performed in 3 groups of cells treated with the optimal concentration of antagomiR-21: SK-N-SH/WT, SK-N-SH/FTH1 and SK-N-SH/FTH1-3×C_miR-21 cells. A significant signal reduction was observed in only the SK-N-SH/FTH1-3×C_miR-21 cells after antagomiR-21 transfection compared with the signal prior to antagomiR-21 treatment (*P*<0.05). There were no changes in the MRI signals change before and after antagomiR-21 treatment in the other two groups of cells (**Figures 5A, B**).

The Prussian blue staining and TEM results were consistent with the MRI findings. Before transfection of antagomir-21, large amounts of iron particles were detected in the cytoplasms of only SK-N-SH/FTH1 cells, while no iron accumulation was observed in SK-N-SH/WT or SK-N-SH/FTH1-3×C_miR-21 cells. After antagomiR-21 treatment, SK-N-SH/FTH1-3×C_miR-21 cells exhibited obvious iron accumulation compared with that prior to antagomiR-21 treatment. In the other two groups of cells, however, there was no change in iron accumulation before and after antagomiR-21 treatment (**Figures 5C, D**). Quantification of the intracellular iron content (**Figure 5E**) showed that the iron content in the SK-N-SH/FTH1-3×C_miR-21 cells was 0.96 ± 0.17 pg/cell after antagomiR-21 treatment, significantly higher than that before antagomiR-21 treatment (0.12 ± 0.02 pg/cell) (*P <*0.05). There was no difference of iron content before and after antagomiR-21 treatment in the other two groups.

The above results suggest that miR-21 in SK-N-SH/FTH1-3×C_miR-21 cells can be inhibited by antagomiR-21 and reboot the expression of FTH1, thereby resulting in intracellular iron accumulation. This process can be detected by MRI signal changes.

### *In Vivo* MRI of AntigomiR-21 Delivery

**Figure 6:**
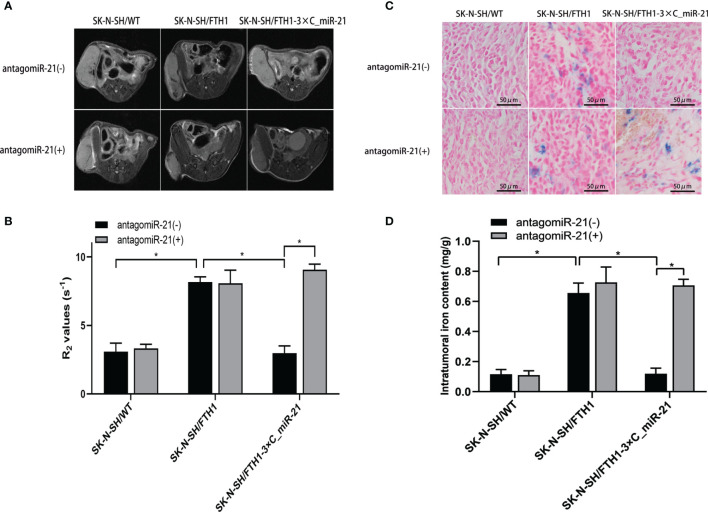
MRI and iron detection in xenografts before and after antagomiR-21 administration. Before antagomiR-21 administration, the MRI signal in the SK-N-SH/FTH1 group was obviously decreased and the R2 value was significantly increased compared with those in the SK-N-SH/WT group, but these values in the SK-N-SH/FTH1-3×C_miR-21 group did not differ from those in the SK-N-SH/WT group. After treatment with the antagomiR-21, the SK-N-SH/FTH1-3×C_miR-21 group showed a significantly decreased MRI signal and increased R2 value compared with those prior to treatment. In the other two groups, the MRI signals and R2 values did not differ before and after antagomiR-21 treatment **(A, B)**. The Prussian blue staining **(C)** and intratumoral iron content quantification **(D)** results match the MRI results. Intratumoral iron accumulation was significantly increased after antagomiR-21 treatment in only the SK-N-SH/FTH1-3×C_miR-21 group. Three independent experiments were performed. * indicates *P* < 0.05.

Animal tumor models of three SK-N-SH cell lines were successfully established. Before administration of the GO-PEG-dendrimer/antagomiR-21 complex, the MRI signal was obviously decreased and the R2 value was significantly increased in the SK-N-SH/FTH1 group (*P*<0.05) compared with the SK-N-SH/WT group, while those in the SK-N-SH/FTH1-3×C_miR-21 group did not differ from those in the SK-N-SH/WT group. After treatment with the complex, the SK-N-SH/FTH1-3×C_miR-21 group showed a significantly decreased MRI signal and increased R2 value compared with those prior to treatment (*P*<0.05). In the other two groups, the MRI signals and R2 values did not differ before and after antagomiR-21 treatment (**Figures 6A, B**).

Further intratumoral iron detection and quantification matched the MRI results. Intratumoral iron content was significantly increased after antagomiR-21 treatment in only the SK-N-SH/FTH1-3×C_miR-21 group compared with that prior to treatment (**Figures 6C, D**).

## Discussion

Increasing evidence has demonstrated that miRs are involved in tumorigenesis and tumor progression *in vitro* and *in vivo*, which provides a new pathway for exploring the potential of miRs as biomarkers for tumor diagnosis and/or therapy (2, 3, 15, 37). In this study, we designed a novel gene reporter system which contained three copies of the sequence completely complimentary to miR-21 in the 3’ UTR of FTH1 for the MRI visualization of miR-21 in neuroblastoma. The results showed that the miR-21-responsive reporter gene was sensitively regulated by miR-21 in neuroblastoma cells. Compared with the regular FTH1 reporter gene, the miR-21-responsive reporter gene was “switched off” by the combination of intratumoral miR-21 and the 3’ UTR of FTH1. AntagomiR-21 induced exogenously “switched on” FTH1 expression in the cells by binding to miR-21 and thereby promoting its release from the FTH1 3’ UTR. FTH1 expression induced a sufficient amount of intracellular iron accumulation for visualization by MRI *in vitro* and *in vivo*. In addition, the reporter gene expression reversed by antagomiR-21 was dose-dependent, suggesting that miR-21 activity can be quantified by MRI. To the best of our knowledge, this is the first report of using an MRI-based gene reporter system to study miR-21 activity in tumors. Our research provides a potential noninvasive imaging method for evaluating miR-21 as a diagnostic or therapeutic target.

Recently, optical imaging techniques based on luciferase or bioluminescent reporter genes have been applied in the miR research field and been shown to serve as noninvasive methods for detecting and quantifying miRs *in vivo*. Ko et al. (38) developed a reporter gene system, CMV/Gluc/3×PT-miR-124, and transduced it into stem cells, and the results showed miR-124a-dependent Gluc reporter expression during neural differentiation. Kim et al. (39) utilized a CMV/Gluc-3xPT_miR221 reporter system to image miR-221 in papillary thyroid carcinoma and showed that Gluc activity was regulated according to the miR-221 levels *in vitro* and *in vivo*. These optical imaging reporter gene systems have greatly expanded our understanding of miR activities *in vivo*. However, optical imaging modalities can be used only for studies in small animals or surface tissues due to their poor tissue penetration, which has limited their clinical application. In the present study, we utilized MRI to detect miR-21 activity and achieved results consistent with those of optical imaging studies. In addition, compared with optical imaging, MRI can better penetrate deep tissue, which may make it more applicable in the miR imaging research field and more feasible for clinical translation.

Employing antisense oligonucleotides to antagonize the functions of target miRs *in vivo* has been proposed as a potential gene therapy for various diseases (40–42). This therapeutic strategy, however, has largely been impeded due to the lack of effective gene delivery carriers. In this study, we constructed a polyamidoamine dendrimer and PEG-functionalized GO conjugate for the delivery of antagomiR-21 into SK-N-SH cells. The efficiency of the GO-PEG-dendrimer to deliver antagomiR-21 was affected by the GO/PEG ratio. Among the three GO/PEG ratio groups, the GO-PEG dendrimer with a GO/PEG ratio of 1:1 delivered antagomiR-21 more efficiently than the commonly used transfection agent siRNA-Mate. The other two structures, which had GO/PEG ratios of 5:1 and 1:5, had delivery efficiencies that were similar to that of siRNA-Mate. We speculate that the decreased transfection efficiency of the GO-PEG-dendrimer with a GO/PEG ratio of 5:1 was due to the decreased biocompatibility of GO-PEG dendrimers. The lower transfection efficiency of the GO-PEG-dendrimer with a GO/PEG ratio of 1:5 may be explained by the reduced capacity of the GO to combine with antagomir-21. Based on the optimal GO/PEG ratio, we further compared the cytotoxicities of GO-PEG-dendrimer and siRNA-Mate. The cell viability of the SK-N-SH cells transfected with the GO-PEG-dendrimer was higher than that of the cells transfected with siRNA-Mate during 72 h of continuous culture, suggesting that the GO-PEG-dendrimer is a safe gene delivery vector. In addition, the *in vitro* and *in vivo* experiments revealed that the antagomiR-21 delivered by the GO-PEG-dendrimer definitely induced FTH1 expression by antagonizing miR-21, thereby indirectly confirming the GO-PEG-dendrimer efficiency as a gene delivery vector.

Although the miR-21-responsive FTH1 gene reporter system was proven to have potential for the MRI assessment of miR-21 function in tumor cells, there are several drawbacks in this study. First, the reporter gene system was transduced into the tumor cells in advance to evaluate the activity of miR-21, and this method is limited to experimental studies and is not feasible for clinical applications. To use this gene reporter system to detect intracellular miR-21 for diagnostic purposes in humans, the system should be delivered into tumor cells by an efficient gene carrier to examine the results in real time. Second, to increase reporter gene expression-induced intracellular iron uptake to a level that is sufficient to generate MRI contrast *in vivo*, exogenous iron was administered to the animal at a certain concentration. At present, it could be difficult to produce an obvious MRI signal change depending merely on endogenous iron recruitment. With the development of MRI equipment and technology, we believe that the endogenous iron concentration would provide enough the intracellular iron accumulation for MRI contrast production, which are needed to explore in further studies. Third, the MRI examination was performed only once after antagomir-21 delivery *in vivo* in consideration of animal welfare and fast tumor necrotization. A more suitable tumor xenograft model should be established to allow MRI examinations at more time points, which may enable a longitudinal MRI monitoring study of miR functions.

## Conclusion

In summary, we developed a novel miR-21-responsive gene reporter system for the MRI visualization of miR-21 dynamics in neuroblastoma. In the presence of active miR-21 in tumor cells, the reporter gene expression was in an “off” state due to the specific combination of miR-21 with 3 copies of completely complimentary sequences (3xC_miR-21) at the 3’ UTR of the reporter gene. The exogenous delivery of antagomiR-21 into the cells by the GO-PEG-dendrimer switched “on” the reporter gene expression because miR-21 bound with antagomiR-21 in a competitive manner and was thus released from the FTH1 3’ UTR. Reporter gene expression resulted in intracellular iron accumulation, thereby allowing MRI visualization. This study provides a noninvasive and feasible imaging method for the detection of miR-21, which is regarded as a potential diagnostic and/or therapeutic target in tumors.

## Data Availability Statement

The original contributions presented in the study are included in the article/**Supplementary Materials**. Further inquiries can be directed to the corresponding author.

## Ethics Statement

The animal study was reviewed and approved by Animal Care and Use Committee of Chongqing Medical University.

## Author Contributions

GB and JC supervised the research. All authors were involved in the design of the study or the acquisition and analysis of the data. GB, JS, JHou, HZ, YF, XZ, and BX performed related experiments. GB, JS, HZ, JQ, JHua, and YF analyzed the data and prepared the figures. GB, JS, and JC wrote the main manuscript. GB, JS, and HZ contributed equally to this work. All authors contributed to the article and approved the submitted version.

## Funding

This study was supported by a grant from the National Natural Science Foundation of China (no. 81771892).

## Conflict of Interest

The authors declare that the research was conducted in the absence of any commercial or financial relationships that could be construed as a potential conflict of interest.

## Publisher’s Note

All claims expressed in this article are solely those of the authors and do not necessarily represent those of their affiliated organizations, or those of the publisher, the editors and the reviewers. Any product that may be evaluated in this article, or claim that may be made by its manufacturer, is not guaranteed or endorsed by the publisher.

## Abbreviations

CCK-8, Cell Counting Kit-8; FTH1, Ferritin heavy chain 1; DMEM, Dulbecco’s modified Eagle’s medium; FAC, Ferric ammonium citrate; FBS, Fetal bovine serum; FOV, Field of view; GO, Graphene oxide; LV, Lentiviral vector; miRs, MicroRNAs; MRI, Magnetic resonance imaging; mRNAs, Messenger RNAs; PBS, Phosphate-buffered saline; PCR, Polymerase chain reaction; PEG, Polyethylene glycol; qRT-PCR, Quantitative reverse-transcriptase polymerase chain reaction; 3’ UTRs, 3’-untranslated regions; 3xC_miR-21, 3 copies of the sequence completely complementary to miR-21; T2WI, T2-weighted imaging; TE, Time of echo; TEM, Transmission electron microscopy; TR, Time of repetition; WB, Western blotting.
