# Supplementary material for: Visualization of microRNA-21 Dynamics in Neuroblastoma Using Magnetic Resonance Imaging Based on a microRNA-21-Responsive Reporter Gene
Source: Front Oncol. 2021 Nov 5;11:747305. doi: 10.3389/fonc.2021.747305 (PMC8602822; doi:10.3389/fonc.2021.747305)
Supplement: Supplementary file 1 [file DataSheet_1.pdf]

Synthesis and characterization of the GO-PEG-dendrimer

1 Methods

The detailed protocol for synthesizing GO-PEG-dendrimer was as follows. First, 0.025 g of GO was dispersed into 50 mL of distilled water and sonicated for 1 h to obtain solution A. Moreover, 0.025 g of six-armed amine-functionalized PEG (6ARM-PEG10000-NH2, MW: 10000, Ponsure) was added to 50 mL of distilled water to obtain solution B. Solution B was rapidly added to solution A and stirred for 5 min at 25 ℃. After that, 0.025 g of N-(3-(dimethylamino) propyl)-N'-carbodiimide hydrochloride (DC, Sigma) and 0.5 g of the dendrimer solution (2 mg/mL, generation 3.0 solution, Sigma) were added. After stirring for 3 h, 0.05 g of DC was added and continuously stirred for 3 h.

Fourier transform infrared spectroscopy (FT-IR) was carried out on KBr disks using a Thermo Fisher Scientific Nicolet iS50 from 4000 to 400 cm-1. X-ray diffraction (XRD) patterns were collected by a PANalytical X'Pert powder. X-ray photoelectron spectroscopy (XPS) was performed on a Thermo Scientific K-Alpha instrument. Zeta potential was determined by a Brookhaven NanoBrook Omni instrument. The sample morphology and thickness were observed on an Asylum Research MFP-3D-BIO atomic force microscope (AFM).

2 Results

GO-PEG-dendrimer was synthesized by functionalizing GO with NH2-PEG and dendrimer (Figure S1). As shown in Figure S2A, the GO FT-IR spectrum exhibited obvious characteristic peaks of C=O, C-OH, C-O-C, O-H, and C=C at 1725.6, 1409.7, 1038.1, 3202.7, and 1622.1 cm-1, respectively, thereby illuminating the presence of -COOH, C=O, -OH, and C-O-C. After modification by NH2-PEG and dendrimer, the characteristic peak of C=O at 1725.6 cm-1 shifted to 1643.0 cm-1, indicating the formation of -NH-CO-. In addition, other peaks also shifted, and some new peaks were exhibited. These results fully demonstrate that GO was successfully functionalized by NH2-PEG and dendrimer. XRD technology was employed to detect changes in the layer spacing of GO after modification. Figure S2B shows that the GO-PEG-dendrimer had obviously different diffraction patterns from GO. After modification, the 2θ peak of GO at 10.8° shifted to 10°. According to the Bragg equation $2d\sin\theta=n\lambda$, larger θ values are correlated with smaller d values. Thus, the layer spacing of the GO-PEG-dendrimer was increased, further proving the conclusion of FT-IR. In addition, a new peak at 2θ=22.3° was observed, and this feature, which illustrates the reduction of GO, has been proven in a previous study. Zeta potential analysis showed that the GO-PEG dendrimer had a positive average zeta potential of 5.5 mV, implying that its surface contained abundant positive charges, which is beneficial for loading other negatively charged molecules (Figure S2C). Content analysis using XPS technology demonstrated that after modification, the O content of GO decreased, and many N elements were introduced. Moreover, the GO and GO-PEG dendrimer films formed under the same conditions had apparently diverse roughness and thickness attributes (Figure S2D). As shown in Figure S3, the thickness of the GO-PEG-dendrimer film was higher than that of the GO film. Therefore, all of these results fully indicate successful GO-PEG dendrimer synthesis.Supplementary Figures

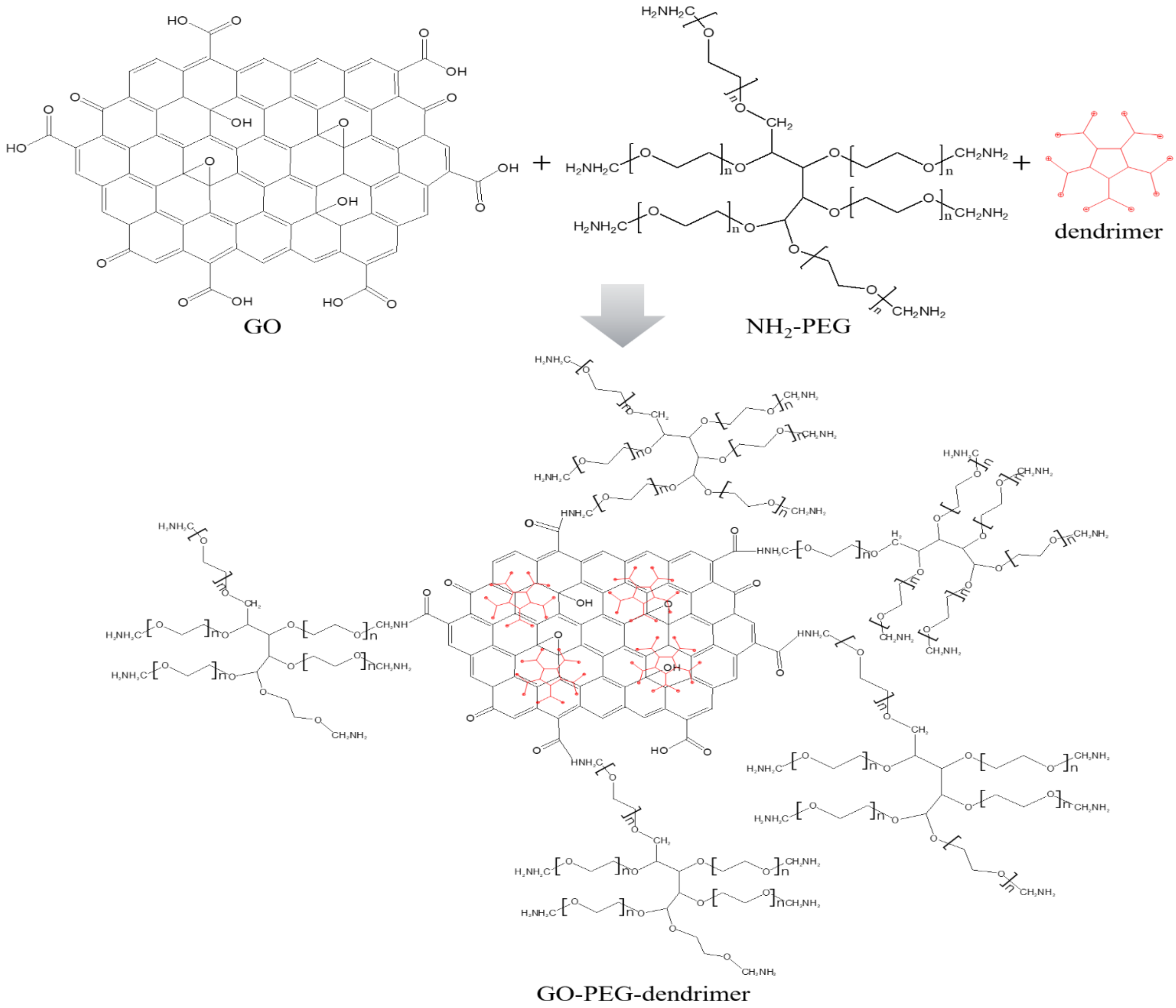

Figure S1. GO-PEG-dendrimer synthesis.

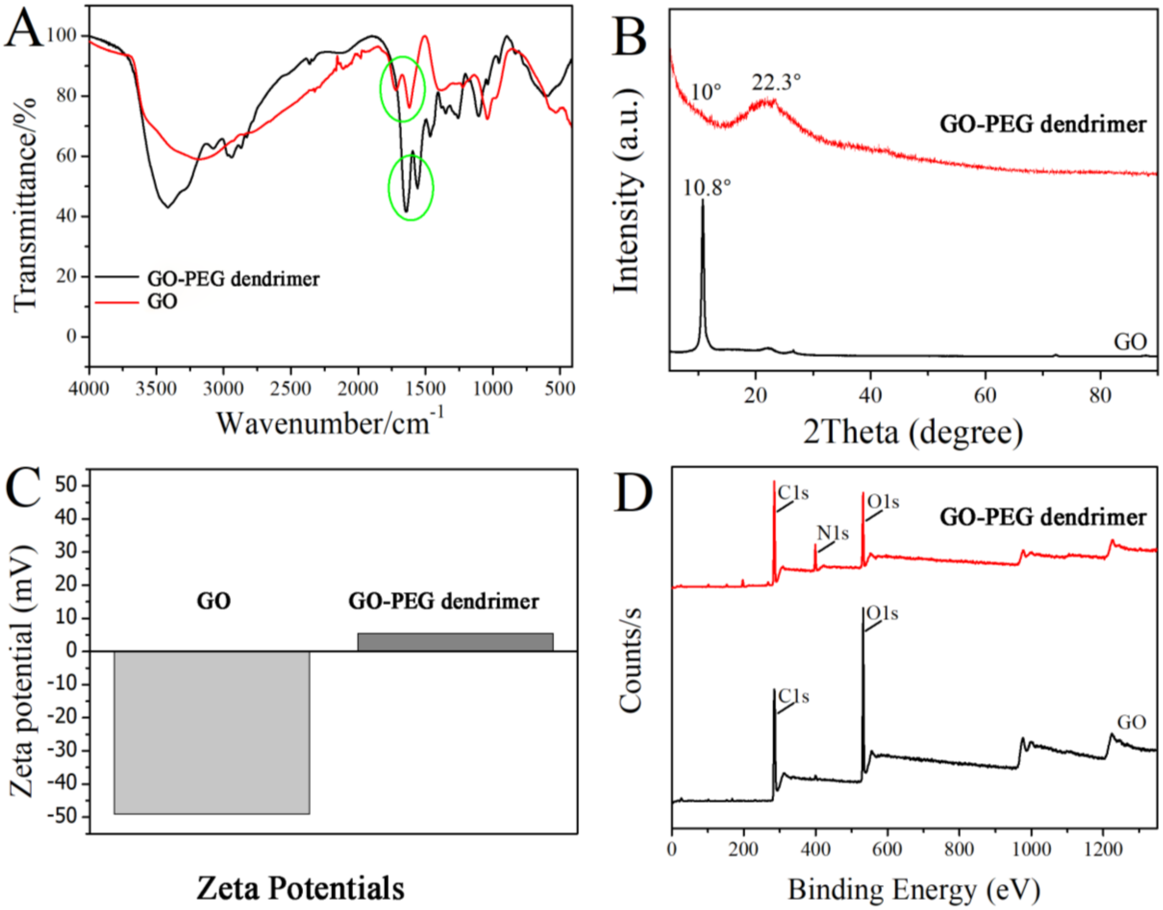

Figure S2. (A) GO and GO-PEG-dendrimer FT-IR spectra. (B) GO and GO-PEG endrimer XRD patterns. (C) GO and GO-PEG dendrimer zeta potentials. (D) GO and GO-PEG endrimer XPS surveys.

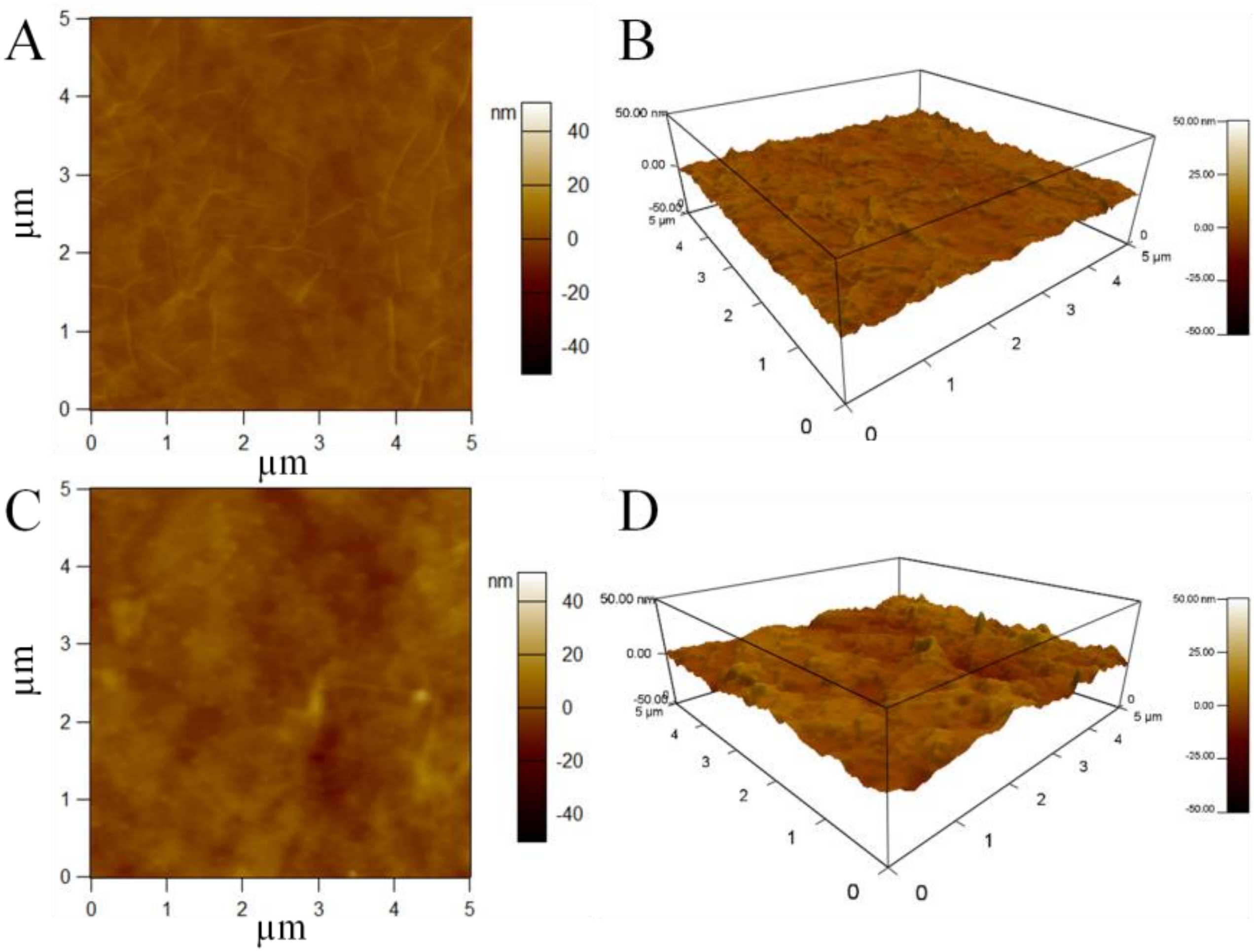

Figure S3. (A and B) AFM images of GO. (C and D) AFM images of the GO-PEG dendrimer.
